# Dog Bite Risk: An Assessment of Child Temperament and Child-Dog Interactions

**DOI:** 10.3390/ijerph9083002

**Published:** 2012-08-20

**Authors:** Aaron L. Davis, David C. Schwebel, Barbara A. Morrongiello, Julia Stewart, Melissa Bell

**Affiliations:** 1 Department of Psychology, University of Alabama at Birmingham, 1300 University Blvd, CH 415, Birmingham, AL 35294, USA; Email: schwebel@uab.edu; 2 Department of Psychology, University of Guelph, 50 Stone Road East, Guelph, ON N1G 2W1, Canada; Email: bmorrong@uoguelph.ca (B.A.M.); jstewa02@uoguelph.ca (J.S.); mbell01@uoguelph.ca (M.B.)

**Keywords:** temperament, injury, dog bites, children, shyness

## Abstract

Annually approximately 400,000 American children receive treatment for dog bites. Young children are at greatest risk and are frequently bitten following behavior that provokes familiar dogs. This study investigated the effects of child temperament on children’s interaction with dogs. Eighty-eight children aged 3.5–6 years interacted with a live dog. Dog and child behaviors were assessed through observational coding. Four child temperament constructs—impulsivity, inhibitory control, approach and shyness—were assessed via the parent-report Children’s Behavioral Questionnaire. Less shy children took greater risks with the dog, even after controlling for child and dog characteristics. No other temperament traits were associated with risk-taking with the dog. Based on these results, children’s behavior with unfamiliar dogs may parallel behavior with other novel or uncertain situations. Implications for dog bite intervention programs include targeting at-risk children and merging child- and parent-oriented interventions with existing programs geared toward the physical environment and the dog.

## 1. Introduction

Approximately 377,000 American children receive medical attention for dog bites every year [1], and about sixteen die [2]. Although fatalities from dog bites are rare, there is a significant risk of injury. Most vulnerable are young children, partly because of their unintentionally provocative behavior with dogs [3] and partly because their short stature results in bites to the face and neck rather than to extremities [4]. Most bites to children are from familiar dogs; in fact, 78% of dog bites to young children occur in the home [5].

One reason children under the age of 5 are bitten by familiar dogs is because the children unknowingly provoke the animals [3,6]. Developmental psychologists have long recognized that young children have a poor understanding of the perspectives of other people [7] and therefore are unlikely to recognize the emotions or behavioral signals of dogs provoked by unwanted child behaviors [8]. For example, in one study of the interactions of children aged 2–5 years with the family dog, children frequently initiated risky interactions with the pets by pulling dogs’ tails, hair or paws. On nearly one-third of such occasions, the dogs bit or attempted to bite the children [9]. Also placing young children at heightened risk of dog bites are children’s natural tendencies to be active, loud and excitable, and to be protective of property. Running and screaming can scare and anger dogs. Child-dog conflicts over toys and other property can lead to biting incidents [3]. Further, although dogs have some ability to identify familiar faces [10], dogs obviously do not have cognitive skills of humans and are unable to recognize that children may be more provocative or animated than adults.

Although some researchers have suggested environmental circumstances that might lead dogs to bite young children [11,12], relatively little research has examined individual difference characteristics of children that might increase the risk for dog bites. There is some epidemiological evidence that boys are bitten slightly more often than girls [12,13,14], although one study found no significant difference in dog bites between boys and girls in The Netherlands [4]. Research has also shown that preschool-aged and school-aged children have somewhat elevated risk compared to infants, toddlers, and teenagers [13,14,15]. One factor that has not been studied carefully as a risk factor for dog bites is child temperament, or individual differences in reactivity and self-regulation [16]. A large body of research indicates children who are more impulsive, active and undercontrolled are likely to be at increased risk for injuries of all types [17], presumably due to behavior patterns of risk-taking, failing to inhibit impulses and engaging in the environment more actively.

The current study considered four temperament traits—impulsivity, inhibitory control, approach, and shyness—as possible correlates to children’s risky behavior with dogs. These traits were assessed using the parent-report Children’s Behavioral Questionnaire (detailed below). Eight-eight children aged 3.5 to 6 years interacted with an unfamiliar live dog for 15 minutes in a mixed (partly unstructured, partly semi-structured) laboratory protocol. Child and dog behaviors were videotaped, coded and then related to parental reports of children’s temperament using a standardized questionnaire. We hypothesized that children who had high impulsivity, low inhibitory control, low shyness, and high approach would be more likely to approach a dog more quickly and to take more risks when interacting with an unfamiliar dog. 

## 2. Method

### 2.1. Participants

Participants in this two-site study were 88 children ages 3.5–6 (mean age = 4.85 years, *SD* = 0.90). Participants were recruited from community sources, including laboratory databases of families interested in participating in research and via local schools. Because dog bite risk is much higher among children who frequently encounter dogs, the sample was restricted to children whose families owned between one and three pet dogs. Participants were recruited from two locations. Sixty-three children were recruited from the Birmingham, Alabama, USA area and 25 from Guelph, Ontario, Canada. Identical procedures were used at both study sites.

The sample was 46% male and 76% Caucasian. Household incomes were characterized as follows: 63% greater than $80,000, 14% between $40,000 and $79,000, and 23% less than $39,000 (US and Canadian currencies were priced very similarly at the time of the study). All parents provided signed informed consent indicating that the child would be engaging with a live certified therapy dog and all children age-appropriate verbal assent acknowledging they would be interacting with a dog. The study protocol was approved by the ethics review boards at both University of Alabama at Birmingham and University of Guelph.

### 2.2. Live Dog Interaction Protocol

As detailed below, children engaged with a certified therapy dog in a protocol that included both semi-structured and unstructured segments. All dogs and handlers had received certification in either the Delta Society Pet Partners or Canine Good Citizens programs. Dog breeds (and shapes/sizes) varied widely and included Cavalier King Charles Spaniels, Giant Poodles, Labrador Retrievers, and Greyhounds. Dogs and children were always unfamiliar to each other (that is, the dogs were not the children’s pet dogs), and children were not informed of the interaction with the dog until the informed assent process on the day of the interaction, just before the interaction occurred. The interaction was videotaped for later coding of dog and child behaviors; details of the coding system appear below.

**Figure 1:**
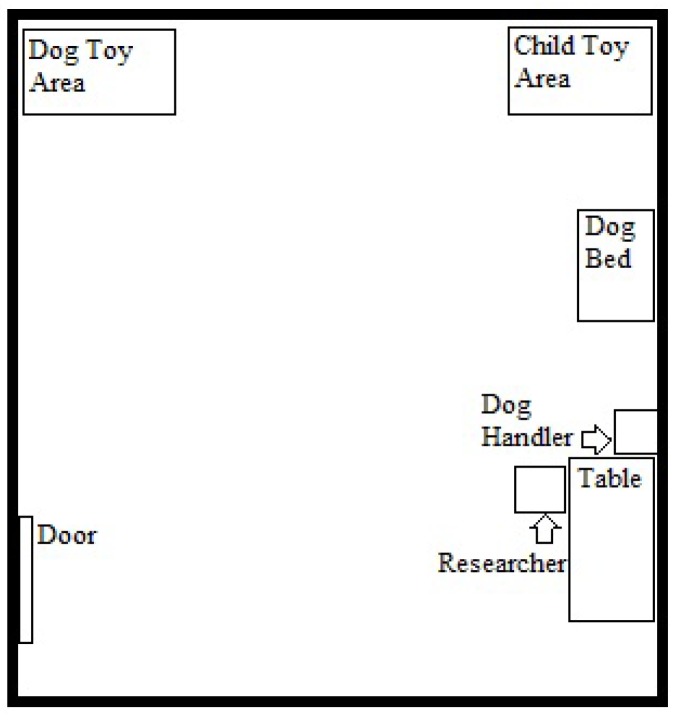
Diagram of Dog Interaction Room.

The full dog-child interaction lasted 15 minutes and consisted of four segments. The first 3-minute segment was unstructured. The child entered the experimental room with the researcher, the live dog, and the dog’s handler, but without his or her parent. Figure 1 provides a diagram of the room layout. It was arranged with a small number of rather mundane child toys in one corner, a small number of dog toys in a second corner, and a dog bed along one wall. Each set of toys was neatly organized in a pile. The researcher said, “*This is [dog’s name]. He’s [She’s] visiting our lab today. [Handler’s name] and I have to do a little work for a few minutes, so you can just do what you like while we work. There are some toys here for children. Those over there are the dog’s toys.*” The handler stayed quiet and remained in the corner of the room, verbally or physically intervening only if he/she felt the dog or child might be at risk of injury (interventions were extremely rare, occurring only for two children, one who pulled the dog’s fur and one who tried to lift a large dog up in the air). 

The second segment was semi-structured and lasted 1–2 minutes. The researcher said, 

*“OK, our work is done now. [Dog’s name] is ready to play. You can play with the dog using one of these things. This is a ball and that is a brush and that is a bowl with three dog treats. If you choose the ball, you can stand over there and throw it to the dog. He[she] likes to retrieve balls. If you choose the brush, you can stand here, right next to the dog, and brush his[her] hair. He[she] likes that too. If you choose the treats, you should hold them in your palm like this [researcher demonstrates open palm] and let the dog lick them off your hand.”*

The child chose one of the three options and engaged in the activity under researcher supervision.

The third segment lasted 3 minutes and was unstructured. The child was told “*Oh, look what time it is. The dog likes to rest at this time. You can do whatever you want for a few minutes while the dog rests and I finish my work with [handler’s name].*” During the fourth segment, which was also unstructured, the child’s parent entered the room. The child, dog and parent spent 5 minutes engaged in activities. Because parents tended to control children’s activities and behaviors during this segment and our focus was on the individual child’s temperament without parent influence, activities during this segment were not included in our coding.

### 2.3. Measures

Demographics*.* Parents completed a brief demographics survey assessing basic child and family demographics.

Child Risk-Taking during Dog Interaction. Videotapes of children’s behaviors during the interaction with the live dog were coded by a research assistant to assess a single construct of children’s risk-taking with the dog via multiple behavioral measures. The research assistant was trained by the principal investigator of this study, and interrater reliability was obtained through independent post-training coding of videotapes by both the principal investigator and the research assistant. Interrater reliability on coding behaviors was high (*r* > 0.95 on all continuous measures and kappa > 0.80 on all categorical measures). Differences were resolved by using data from the primary coder, who rated all participants. 

Risk-taking was conceptualized as a behavior pattern of approaching, touching and interacting with the dog when the outcome of that behavior was uncertain [18]. We considered behaviors that were bold, brave, or assertive, but not necessarily behaviors that would typically lead to provocation of the dog or to dog bites. Table 1 shows the behaviors that were coded to derive the measure. Categorical variables were assigned ordinal numbers to represent increasing risk-taking. In order to create the single aggregated risk-taking measure for each child’s interaction with the dog, the variables were each converted to z-scores and then averaged. Internal reliability for the scale was good (average intercorrelation = 0.44; Cronbach’s alpha = 0.73).

**Table 1:** Children’s Coded Behaviors during Live Dog Interaction.

1. What does child touch first after entering room? (Segment 1)
1. Child Toys; 2. Dog Toys; 3. Dog
2. Which activity does child choose? (Segment 2)
1. Ball; 2. Brush; 3. Treat
3. Does child pick up a dog toy? (Segments 1, 3)
1. No; 2. Yes
4. How long until child engages the dog? (in seconds) (full protocol)
5. How does child engage dog for the first time? (Segments 1, 3)
1. Throws toy to dog; 2. Calls dog to him/her; 3. Approaches dog;
4. Touches dog after dog approaches child
6. How long until child approaches dog 1st time? (in seconds) (full protocol)
7. How does child first approach dog? (full protocol)
1. Hesitantly/Cautiously; 2. Approaches and then backs away;
3. Quickly/Unhesitatingly approaches
8. How long until child touches the dog for the 1st time? (in seconds) (full protocol)
9. How does child first touch dog? (full protocol)
1. Hesitantly/Cautiously; 2. Touches then withdraws; 3. Quickly/Unhesitatingly
10. Number of times child touches the dog with hand. (full protocol)
11. Number of times child put their face to the dog’s face. (full protocol)
12. Child’s approach during “nap.” (Segment 3)
1. Looks at dog; 2. Gets within arm’s reach of dog; 3. Touches dog
13. Child’s reaction to dog’s approach during free play. (Segment 1)
1. Runs From Dog; 2. Freezing/Cautious; 3. Tells dog to go away;
4. Smiling/Laughing; 5. Pushes Dog away
14. Child’s reaction to dog’s approach during activity. (Segment 2)
1. Runs From Dog; 2. Freezing/Cautious; 3. Tells dog to go away;
4. Smiling/Laughing; 5. Petting/Touching Dog; 6. Pushes Dog away
15. Child’s reaction to dog’s approach during nap time. (Segment 3)
1. Runs From Dog; 2. Freezing/Cautious; 3. Tells dog to go away;
4. Smiling/Laughing; 5. Petting/Touching Dog; 6. Pushes Dog away; 7. NA

Dog Behavior*.* Because different children engaged with different dogs, we coded dog behavior and size from the videotapes as covariate measures. The same two coders independently rated 20% of the sample using objective coding criteria and achieved strong interrater reliability (kappa > 0.80) on all measures. The primary coder subsequently coded the full sample and data from the primary coder were used.

Dog size was coded on a 4-point scale as small (less than 25 pounds), medium (25–50 pounds), large (51–75 pounds), or very large (75+ pounds). The dogs’ behavior was coded in two domains, activity level and level of approach. In each case, the constructs were assessed on 5-point Likert scales ranging from low to high during each of the three relevant segments of the interaction (See Table 2). Scores from the three segments were averaged to obtain one overall measure of dog activity level and one of dog approach for each child’s interaction with the dog. Then, to create a single variable for the dog’s behavior during the interaction, the average of the dog’s activity level and approach were each converted to z-scores and then averaged, creating a single score for the dog’s behavior. Activity level and approach correlated well, *r* = 0.67.

**Table 2:** Dog’s Coded Activity Level and Approach during Each Segment of the Interaction.

Dog’s activity level
1. Very inactive (Moves around room very rarely)
2. Somewhat inactive (Moves some, but spends majority of time not moving)
3. Not inactive or active (Spends about equal amounts of time moving and not moving)
4. Somewhat active (Does not move some, but spends majority of time moving around room)
5. Very active (Moving around room all or almost all the time)
Level of approach for dog (approach defined as: DOG moves or positions itself within child’s arm’s length of the child)
1. Low approach (Never approaches child)
2. Somewhat low approach (Only approaches child once)
3. Neither low nor high approach (Approaches child 2–3 times)
4. Somewhat high approach (Approaches child 4–6 times)
5. High approach (Approaches child 7 or more times)

Child Temperament*.* Parents completed four subscales–impulsivity, inhibitory control, shyness, and approach–from the Children’s Behavior Questionnaire (CBQ) [19], a standard parent-report measure of child temperament designed for use with children ages 3 to 7. Each subscale is comprised of 13 items, and all items are answered by parents on a 7-point Likert scale ranging from extremely untrue to extremely true. Impulsivity is defined as the speed of response initiation; a sample item assessing impulsivity is, “My child usually rushes into an activity without thinking about it.” Inhibitory control is defined as the capacity to plan and to suppress inappropriate responses under instructions in novel or uncertain situations. A sample item assessing inhibitory control is, “My child can lower his/her voice when asked to do so.” Shyness is defined as slow or inhibited approach in situations involving novelty or uncertainty, and is exemplified by this sample item, “My child sometimes prefers to watch rather than join other children playing.” Approach is defined as the amount of excitement and positive anticipation for expected pleasurable activities. A sample item assessing approach is, “My child gets so worked up before an exciting event that s/he has trouble sitting still” [20]. Internal reliability of all four scales is good, Cronbach’s alphas ranging from 0.74–0.94 [19].

### 2.4. Data Analysis Plan

Data analyses were completed in three steps: (a) descriptive statistics; (b) Pearson correlations between risk-taking and the four temperament traits (impulsivity, inhibitory control, shyness, approach); and (c) linear regression models to predict risk-taking by temperament after controlling for relevant dog-specific and child-specific covariates. 

## 3. Results

Descriptive data are presented in Table 3. We considered bivariate correlations between the four temperament traits of interest, impulsivity, inhibitory control, approach and shyness, and risk-taking with the live dog. As shown in Table 4, the four temperament traits tended to intercorrelate with each other, although shyness was not closely related to inhibitory control or approach. Our focus for this study was how the four temperament traits correlated to risk-taking with the dog. No significant correlations were found between risk-taking and impulsivity (*r* = 0.16), inhibitory control (*r* = −0.12), or approach (*r* = 0.10). A significant correlation did emerge between risk-taking and shyness (*r* = −0.23, *p* < 0.05), with higher levels of shyness associated with less risk-taking with the dog. 

**Table 3:** Descriptive Statistics for Variables*.*

Variable	M (SD) or %
Age (in years)	4.85 (0.90)
Gender	46% male
Impulsivity (on 7-point scale)	4.44 (0.83)
Approach (on 7-point scale)	5.25 (0.67)
Shyness (on 7-point scale)	3.64 (1.16)
Inhibitory Control (on 7-point scale)	4.82 (0.73)
Standardized Risk-Taking with Live Dog Aggregate (z-score)	−0.03 (0.60)
Dog Size	61% Small [1]
	4% Medium [2]
	35% Large [3]
	0% Very Large [4]
Dog Activity Level	13% Very Inactive [1]
	33% Somewhat Inactive [2]
	47% Not Inactive or Active [3]
	8% Somewhat Active [4]
	0% Very Active [5]
Dog Approach	13% Low Approach [1]
	35% Somewhat Low Approach [2]
	48% Neither High or Low [3]
	5% Somewhat High Approach [4]
	0% High Approach [5]

**Table 4:** Pearson Correlations of Risk-taking and Impulsivity, Inhibitory Control, Approach and Shyness (N = 88).

	2	3	4	5	6	7	8	9
1. Risk-Taking	0.16	−0.01	0.10	−0.23 *	−0.01	0.09	0.16	0.12
2. Impulsivity	-	−0.53 *	0.54 **	−0.63 **	−0.24 *	−0.10	−0.04	−0.03
3. Inhibitory Control		-	0.44 **	0.09	0.16	0.13	−0.10	0.02
4. Approach			-	−0.20	−0.19	−0.07	−0.01	−0.07
5. Shyness				-	0.19	0.06	0.14	−0.08
6. Gender					-	0.10	0.09	0.04
7. Age						-	0.03	−0.15
8. Dog Behavior							-	0.31 *
9. Dog Size								-

* p < 0.05; ** *p* < 0.01.

Given bivariate results, we constructed a hierarchical linear regression model to test the relation between shyness and risk-taking after controlling for potential covariates (See Table 5). Four covariates were included in the first step of the model: child age, child gender, dog size, and dog behavior (*R^2^* = 0.07, *F*(4,87) = 1.56, ns). With shyness included in the second step, all predictors accounted for 13% of the variance in risk-taking, and the overall model was significant (*R^2^* = 0.13, *F*(5,87) = 2.49, *p* < 0.05). The association between shyness and risk-taking remained statistically significant after controlling for all covariates (β = −0.26, *t*(82) = −2.42, *p* < 0.05). The only other significant predictor in the final model was the dog’s behavior during the interaction (β = 0.25, *t*(82) = 2.27, *p* < 0.05), which indicated that the more active/approaching the dog was, the more risk-taking the child showed.

**Table 5:** Summary of Hierarchical Linear Regression Analysis for Variables Predicting Risk-Taking (N = 88).

Variable	*B*	*SE B*	Β
Step 1			
Gender	−0.06	0.13	−0.05
Age	0.08	0.07	0.12
Dog Size	0.13	0.07	0.21
Dog Behavior	0.15	0.05	0.22 *
Step 2			
Gender	−0.01	0.13	−0.01
Age	0.08	0.07	0.12
Dog Size	0.12	0.07	0.19
Dog Behavior	0.17	0.07	0.25 *
Shyness	−0.13	0.06	−0.26 *

* *p* < 0.05.

## 4. Discussion

Temperamental shyness, but not approach, impulsivity, or inhibitory control, was related to children’s risk-taking behavior with a previously unfamiliar live dog. This association was maintained after controlling for various covariates, including children’s age and gender and the dog’s behavior (approach and activity level) and size. In this study, shyness was conceptualized as “slow or inhibited approach in situations involving novelty or uncertainty” [20]. Our findings indicate, therefore, that children may have interpreted the interaction with the live dog as a novel and uncertain encounter. Those children who were rated by their parents as more shy—those rated as more cautious and fearful in novel or uncertain social situations—tended to be somewhat more cautious and fearful in interacting with the dog. Those parents who rated their children to be less shy—whose children were rated as bolder and less fearful in social situations—were somewhat more likely to take risks in interacting with the dog. 

The fact that greater shyness was associated with lower risk-taking with the dog is sensible. It reflects the possibility that risk-taking by young children with dogs, including behaviors that may sometimes provoke the dog into biting the child, may be driven partially by temperamental traits. In particular, such risk-taking may be more likely in children who are not shy, who are bold and outgoing in social situations, and who respond to novel, unfamiliar, or uncertain situations with some degree of disinhibition or approach tendency. Children who are more cautious, inhibited, or shy in such situations may be protected somewhat from dog-bite injury risk, partly because they avoid potentially injurious situations and therefore have reduced exposure to potentially injurious situations. The finding also supports results from Vollrath and colleagues [21], who found that low scores on a measure of shyness were related to children’s exposure to injuries in traffic and recreational environments.

One surprising aspect of our results was the fact that approach, impulsivity, and inhibitory control were all unrelated to risk-taking with the dog. Previous work has linked both impulsivity and inhibitory control to general unintentional injury risk in this age group [17], and all three traits include behavioral tendencies we expected may have related to risk-taking with the dog.

Our results suggest children’s behavior with dogs is driven more by shyness than by tendencies to approach pleasure (approach) [20] or engage quickly in response to stimuli (impulsivity) [20]. The null finding with approach is surprising. Dogs are likely to invoke pleasure in many children, but perhaps less than other stimuli children encounter. Alternatively, children may not have interpreted an unexpected encounter with an unfamiliar dog as pleasurable; it may instead have aroused emotions of caution, fear, or—as our results suggest—shyness. The null result with impulsivity was also surprising. One possible explanation is that impulsivity may play a greater role in environments where potentially injurious decisions are made quickly (e.g., pedestrian settings) than in less time-urgent situations such as an interaction with an unfamiliar dog.

The fact that inhibitory control was not related to children’s behavior with the dog implies children’s behaviors with the dog were not driven by their capacity to suppress responses. Inhibitory control is expressed via behavioral suppression in at least two circumstances. First, it is expressed when danger is recognized. It is possible that young children did not recognize the danger in interacting with unfamiliar dogs, and therefore had no need to suppress approach responses with them (although therapy dogs were used, children were not explicitly told this fact). Second, inhibitory control is expressed when children face a situation they know is prohibited. It may be that most young children with pet dogs do not have prohibitions (e.g., from parents) against playing with unfamiliar dogs when adults are present, and therefore inhibitory control was not associated with their risk-taking in the experiment. Future research might evaluate these and other possibilities.

## 5. Strengths, Limitations, Implications and Conclusions

There are several strengths in this study. While past research has relied on medical records or parent report of dog bite history to assess the risks for dog bites, this study used a laboratory-based observational design to assess children’s actual behavior with a live dog and then code that behavior via videotape, a methodological strategy that may have improved the validity of assessing children’s behavior with a dog. Several limitations should be noted. First, we relied only on parent report to assess child temperament and did not have a behavioral measure. Second, the sample size was rather modest, although statistical power was sufficient (>0.75) to detect medium effect sizes in the regression model. Third, the live dogs used in the protocol were unfamiliar to the children and were certified therapy dogs. Results may not translate to familiar or pet dogs. Further, although children were not explicitly told the dogs were certified therapy dogs (parents were told during consenting), the dog’s calm nature may have been apparent to them. Fourth, we focused our analysis primarily on temperament and demographic factors. Our regression model accounted for only 13% of the variance in children’s behavior, suggesting other factors of interest were not evaluated in this study. Last, our coding scheme was developed specifically for this study and, though based in theory and previous child temperament research, may not have construct validity to detect variance in the constructs of interest.

The study has implications for both future research and for development of dog-bite intervention programs. From a research perspective, future work might consider laboratory-based experimental designs as one means to continue to explore which children have increased risk of dog bites and under which circumstances. We were able to successfully study children’s interactions with live therapy dogs in a controlled setting, offering a unique first-hand perspective of children’s risk-taking behavior with unfamiliar but certified-to-be-calm dogs. From the perspective of intervention development, our findings suggest children’s individual differences may play a role in risk for dog bite injuries. Current dog bite prevention programs tend to focus primarily on changing dogs and their owners via policy and regulations concerning matters such as use of leashes, controls on high-risk breeds, and obedience training. Educational programs for children have also been found to be somewhat successful in children’s behavior around dogs [22]. Our results suggest interventions targeting children might offer a different avenue toward preventing dog bites. Our results do not indicate that only a certain group of children (non-shy ones) are at risk of dog bites, so broad interventions targeting children would be optimal. With limited resources however, our results do suggest that children who are not shy and live in homes with dogs may have somewhat increased risk of bites and therefore might form a logical choice for more intensive adult supervision while playing with the family pet or for more intensive training in dog safety lessons. In the end, combining broad child-oriented interventions with programs and policy targeting dogs and their owners may be the best strategy to reduce dog bite incidence worldwide.
